# Fluorene-9-bisphenol affects the terminal differentiation of mouse embryonic bodies

**DOI:** 10.1016/j.crtox.2023.100133

**Published:** 2023-11-02

**Authors:** Aidan J. McLaughlin, Anthony I. Kaniski, Darena I. Matti, Nicodemus C. Monear, Jessica L. Tischler, Besa Xhabija

**Affiliations:** a College of Arts Sciences and Letters, Department of Natural Science, University of Michigan-Dearborn, Dearborn, MI 48128, United States; b College of Arts Sciences, Department of Natural Science, University of Michigan-Flint, Flint, MI, United States

**Keywords:** Fluorene-9-bisphenol, BHPF, Mouse embryonic stem cells, Terminal differentiation

## Abstract

Fluorene-9-bisphenol (BHPF) has recently attracted interest as it is increasingly used in industrial settings as a substitute for Bisphenol A (BPA). However, the effects of BHPF exposure on embryonic stem cell (ESC) self-renewal, pluripotency, and differentiation remain poorly understood. This study investigates the impacts of BHPF on mouse embryonic stem cells (mESCs) and embryonic bodies (EBs). Our results reveal that BHPF exposure leads to a morphological shift in mESCs, reducing the percentage of dome-shaped colonies and indicating loss of self-renewal and pluripotency. BHPF exposure also appeared to affect the early stages of EB formation and their growth dynamics, with a reduction in EB numbers and an increase in their size. Subsequent gene expression analysis revealed that BHPF exposure led to increased expression of the inflammatory gene Il6, indicating a potential stress response.

Furthermore, BHPF affected the terminal differentiation pathway, modulating the expression of 16 genes associated with distinct cell types, including lymphatic endothelium, keratinocyte epithelium, pancreatic beta cells, macrophages, monocytes, T-cells, neurons, retinal ganglion cells, nephrons proximal tubule cells, and cardiomyocytes. These findings offer insights into the impact of BHPF on ESC biology and suggest potential implications for developmental and neurodegenerative disorders. Future work should focus on elucidating the underlying mechanisms of BHPF-mediated effects on stem cell function. This may offer new perspectives for understanding the health impacts of environmental exposure to BHPF.

## Highlights

• BHPF alters mouse embryonic stem cells’ morphology and self-renewal, reducing alkaline phosphatase-positive colonies.
• BHPF impacts embryonic stem cell differentiation, resulting in larger, fewer embryonic bodies.
• BHPF affects terminal differentiation pathways, altering expression of tissue-specific cell type genes.

## Graphical abstract

**Figure:**
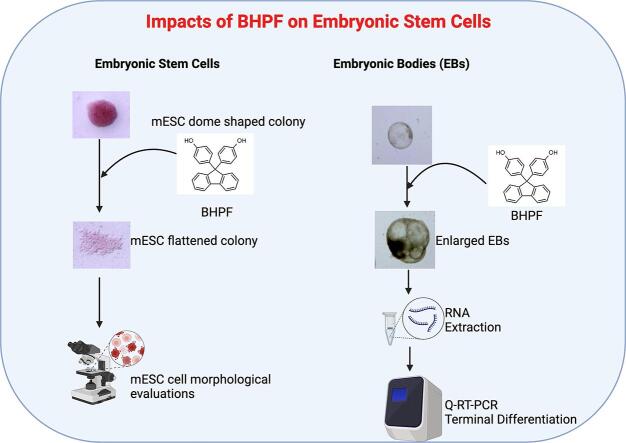

## 1 Introduction

Bisphenols are a group of chemical compounds that have been widely used in the production of polycarbonate and epoxy resins, which are used in many consumer products, including food packaging, medical devices, and electronic equipment since the 1960 s (Moreman et al., 2017, Meng et al., 2019). They constitute a group of synthetic compounds widely utilized in producing polycarbonate plastics and epoxy resins. Bisphenol A (BPA) is the most abundant and has been extensively investigated for its potential adverse impacts on human health and development. BPA can exert endocrine-disrupting effects by mimicking or interfering with the function of natural hormones, particularly estrogen. BPA exposure has been associated with various disorders, such as obesity, diabetes, infertility, reproductive cancers, and neurodevelopmental impairments (Rochester and Bolden, 2015, Han and Hong, 2016, Kim et al., 2019, Mustieles et al., 2020, Pivonello et al., 2020, Sang et al., 2021, Vom Saal and Vandenberg, 2021, Welch et al., 2022). BHPF, a fluorene derivative, has been employed as a 'safer' alternative in BPA-free plastics (Zhang et al., 2017). However, the potential toxicological impact of BHPF is still not fully understood. Moreover, BHPF has been found in household dust, further elevating concerns about widespread exposure (Fan et al., 2023). Due to the growing public concern and regulatory pressure over BPA safety, some BPA alternatives have been introduced, such as BHPF. However, the toxicological profiles of BHPF are largely unknown. Recent studies have suggested that BHPF may similarly have endocrine-disrupting effects like BPA (Huang et al., 2022), and may cause cardiotoxicity. Therefore, there is an urgent need to evaluate the potential developmental toxicity of BHPF.

Previous studies suggest that BHPF may have endocrine-disrupting effects (Huang et al., 2016, Huang et al., 2022) and impaired mouse and human decidualization (Huang et al., 2016, Jin et al., 2022). Beyond its endocrine-disrupting properties, BHPF has been linked to adverse effects on reproductive and developmental health, such as decreased fertility and compromised fetal development (Zhang et al., 2017, Mi et al., 2020). In vitro, experiments further reveal that BHPF negatively affects the production of key hormones, including testosterone, aldosterone, cortisol, and estradiol (Huang et al., 2022). Animal models substantiate these concerns, as BHPF exposure has been linked to reduced birth weight and implantation site weight in pregnant mice (Jin et al., 2022). Moreover, studies indicate that BHPF exposure at a concentration of 1000 nmol/L leads to delayed embryonic development, elevated mortality rates, and altered gene expression, affecting motor neurons and locomotion capabilities (Mi et al., 2019). These studies have been primarily conducted in vitro or animal models. Despite these findings, there is a substantial gap in the literature regarding the specific effects of BHPF on early embryonic development, particularly during the critical post-implantation stages in the uterus.

Embryonic stem cells (ESCs), derived from the inner cell mass of blastocysts, offer a pertinent model system due to their self-renewal capabilities and pluripotency (Amit and Itskovitz-Eldor, 2009). Given existing concerns about BHPF's endocrine-disrupting effects and its ability to impair reproductive and developmental health (Zhang et al., 2017, Mi et al., 2020), we hypothesize that BHPF exposure at concentrations found in human blood serum (Zhang et al., 2017), could adversely affect neurogenesis, stress response, and terminal differentiation in mouse embryonic bodies.

The primary aim of this study is to comprehensively investigate the impact of BHPF on mouse embryonic bodies. Specifically, we aim to evaluate BHPF's effects on neurogenesis, its role in cellular stress and toxicity, and its influence on terminal differentiation. Focusing on concentrations found in human blood serum (Zhang et al., 2017), this study seeks to provide data immediately relevant to human health concerns. The findings from this research could contribute significantly to establishing safe exposure guidelines and deepen our understanding of BHPF's impact on early human development.

## 2 Materials and methods

### 2.1 Cell culture and morphological evaluations

Fluorene-9-bisphenol was purchased from Sigma-Aldrich, 97009, and stocks were stored at − 20C. R1 mouse embryonic stem cells (mESCs) were cultured at 37° C and 5 % CO_2_ humidified atmosphere in DMEM High glucose (Fisher Scientific, 11,965,092) with 15 % ESC grade FBS (Sigma, ES-009-C), 1 % penicillin/streptomycin (Fisher Scientific, SV30010), 1 % ß-mercaptoethanol (Sigma, ES-007-E), 1 mM sodium pyruvate (Fisher Scientific, SH3023901), 1 % non-essential amino acids (HyClone), and 1200 U/ml of leukemia inhibitory factor (LIF) (Sigma, ESG1107) and 1.5 μM CHIR 9901 (GSK3 inhibitor)(Fisher Scientific, NC1039484). Self-renewing mESC colonies are characterized by their round, tightly packed, dome-like morphology, while differentiating mESC colonies display a more spread, flattened appearance, accompanied by decreased alkaline phosphatase activity. To quantify these morphological features and differentiate between self-renewing and differentiating mESC colonies, we employed ZEN lite software and the ZEN software's Interactive Measurement Tool.

Self-renewing mESC colonies are characterized by their round, tightly packed, dome-like morphology, while differentiating mESC colonies display a more spread, flattened appearance, accompanied by decreased alkaline phosphatase activity. Embryonic stem cells (ESCs) are pluripotent cells that can self-renew indefinitely and differentiate into various cell types. ESCs are widely used as in vitro models to study embryonic development and toxicology, as they recapitulate many aspects of early embryogenesis and respond sensitively to environmental cues. In particular, mouse ESCs (mESCs) have been extensively characterized and standardized for developmental toxicity testing (Kang and Jeung, 2019, Lee et al., 2019).

### 2.2 Alkaline phosphatase staining

Given that pluripotent embryonic stem cells exhibit elevated levels of alkaline phosphatase (AP) expression, we employed alkaline phosphatase activity as an indicator of pluripotency. Alterations in this activity serve as potential markers of changes in self-renewal and pluripotency capacity. Following ES cell fixation, a mixture of Fast-Red Violet LB Salt solution (Sigma, 368881) and Naphthol AS Phosphate Solution was applied to the ES cells. We used Image J (https://imagej.nih.gov) to measure the intensity of the alkaline phosphatase stain in 3 independent experiments. We captured ten images per sample and analyzed all mESC colonies using Image J software and Zeiss ZEN Microscopy Software. We changed the images to grayscale and aligned the scale, then used the analyze measure tool to obtain the pixel intensity data, which we converted to percentages. We coated the culture dishes with 0.1 % gelatin water for 15 min before seeding the cells. Zeiss Stemi 508 Stereomicroscopewas utilized to observe the mESC colonies stained with alkaline phosphatase.

### 2.3 Embryonic bodies formation

The differentiation of mouse embryonic stem cells (mESCs) into embryonic bodies (EBs) was induced by removing the Leukemia Inhibitory Factor and CHIR 9901 from the culture medium. To collect the cells as single entities, we employed StemPro accutase (ThermoFisher, A11105) for detachment, followed by resuspension to ensure a single-cell suspension. Prior to detachment, cells were exposed to BHPF for 24 h to investigate the immediate effects of BHPF on mESCs.

Three independent experiments were conducted, each involving the seeding of 200,000 cells per sample in low attachment plates, promoting the formation of three-dimensional EBs. The media was refreshed every 2 days to provide the cells with essential nutrients and remove waste products. Our choice of the static suspension method for EB formation was guided by the known limitations of traditional methods, such as the hanging drop method, regarding scalability and reproducibility (Spelke et al., 2011). Medium exchange using the hanging drop method can disturb the developing embryoid bodies (EBs), potentially affecting their morphology and differentiation. Given these limitations, we opted for the static suspension method, which offers advantages in terms of scalability, reproducibility, ease of manipulation, and handling. This method produces more EBs in a single batch and is more suitable for high-throughput applications (Mogi,Ichikawa et al., 2009).

During the 8-day differentiation period, EBs were incubated in media containing BHPF at concentrations of 0 ng/ml, 0.5 ng/ml, and 1 ng/ml. EBs were exposed to 0.5 ng/ml and 1 ng/ml concentrations to reiterate the blood BHPF concentrations in serum samples from college student volunteers who habitually used plastic bottles for drinking water (Zhang,Hu et al., 2017). For quantitative analysis of EB formation, we utilized Image J software equipped with the cell counter plugin. The initial step involved calibrating the image scale by defining a known distance within the image to ensure that the subsequent measurements were proportionate and accurate. After this calibration, the measurement tool within the software was employed to quantify various parameters associated with the objects in the image, such as area. This process necessitated the user to select the objects of interest within the image. Following this selection, the measurements were readily obtained.

### 2.4 RNA extraction

RNA was harvested from EBs treated with 1 ng/ml BHPF on the eighth day of differentiation using the RNAeasy Kit (Qiagen, 74104). The decision to extract RNA on day 8 was driven by observed morphological disparities, characterized by the larger size of the EBs relative to controls, and the possibility that the EBs had achieved a more advanced stage of differentiation. Three independent experiments were conducted, each encompassing RNA extraction from 2 to 3 embryonic bodies. During RNA isolation, cells were lysed and homogenized in a buffer supplemented with GITC (guanidine thiocyanate) to inhibit RNAase activity. Subsequent ethanol treatment promoted RNA adherence to a silica gel-based membrane column, facilitating total RNA isolation from cellular contaminants. DNA on the columns was degraded with DNAse (Qiagen) before RNA elution.

### 2.5 Quantitative Real-Time RT-PCR

The qRT-PCR was utilized on the RNA extracted from the embryonic bodies 8 days old and exposed to a 1 ng/ml concentration of BHPF. Approximately 500 ng of RNA was reverse transcribed to cDNA using the RT2 First Strand cDNA synthesis kit (Qiagen). Real-time RT-PCR was performed following the manufacturer’s instructions. Three different PCR arrays were utilized.

For gene expression profiling, we utilized the Mouse Terminal Differentiation Markers Pathway PCR Array (PAMM-048Z, Qiagen). We tested one to three specific gene expression markers from 13 major organs or cell types and their subgroups. Genes from adipocytes, chondrocytes, endothelium, epithelium, bone cells, liver cells, bone marrow cells, nervous system cells, retinal cells, nephrons, lung cells, muscle cells, and pancreatic cells were investigated. P-values were calculated based on a Student's *t*-test of the replicate 2 − ΔCT^2^ − ΔCT values for each gene in each control and test group comparison. The statistical method employed for p-value calculation adhered to a parametric, unpaired, two-sample equal variance, and two-tailed distribution approach, as stipulated in the RT^2^ Profiler PCR Arrays & Assays Data Analysis Handbook (Qiagen). Volcano plots were generated to evaluate the gene expression changes in the context of their statistical significance. The plot features the log2 of each gene’s fold change value on the x-axis and the negative log_10_ of each gene’s p-value on the y-axis. The default p-value threshold utilized for the Volcano plot is 0.05. The RT2 Data Analysis tool does not support p-value correction.

For each Gene Ontology (GO) term, the number of differentially expressed (DE) genes annotated to that term is assessed against the number expected to occur merely by chance. Employing an over-representation strategy, iPathwayGuide calculates the statistical significance of observing a given or greater number of DE genes. This significance is determined via the hypergeometric distribution, as outlined for pORA in the Pathway Analysis section, and is subsequently adjusted for multiple comparisons through FDR (Ashburner et al., 2000, Harris et al., 2004). Fifteen genes were considered significant using a fold change cutoff of 2. A Euclidean distance matrix was determined from the Average delta Ct expression of the BHPF treated versus control EBs, and clustering was performed with the complete linkage method. For graphical representation, the R package “pheatmap” was employed.

### 2.6 Statistical evaluation

In our study, a comprehensive statistical analysis was conducted to rigorously assess the significance of observed differences between experimental groups. The Student's *t*-test was applied to compare the replicate 2^−ΔCT^ values of each gene in both the control and treatment groups to evaluate gene expression changes. These 2^−ΔCT^ values represent the normalized expression levels of target genes, calculated using the ΔCT method with an appropriate reference gene as the normalization factor. A p-value threshold of less than 0.05 was set to designate statistical significance, and results meeting this criterion are highlighted in red in the respective figures and tables. Additionally, the *t*-test assumes equal variances and sample sizes for the groups being compared, and these assumptions should be considered when interpreting the results.

## 3 Results

### 3.1 BHPF affects the gross morphological features of the mouse embryonic stem cells (mESC) and mouse embryonic bodies (mEBs)

**Fig. 1:**
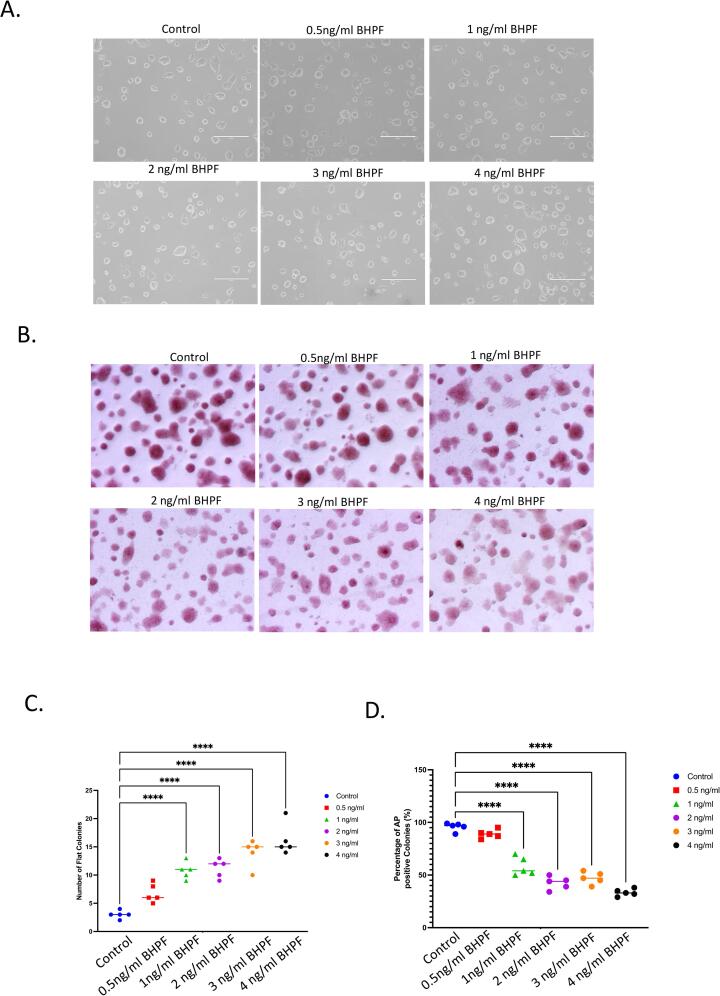
Mouse ESC cells morphological changes following exposure to BHPF **A.** Images of mES cells exposed to 0 ng/ml, 0.5 ng/ml, 1 ng/ml, 2 ng/ml, and 3 ng/ml of BHPF for 24 h. **B.** Alkaline phosphatase staining of mESC cells after 24 h treatments with different concentrations of BHPF. Images were taken with a stereoscope. **C.** Morphological analysis of mESC colonies. The percentage of colonies with embryonic stem cell-like morphology (round and compact) versus non-embryonic stem cell-like morphology (flat or spread out) is shown as mean ± SEM. Each experiment was performed with 5 biological replicates and 10 images per replicate. One-way ANOVA was used to calculate P-values using GraphPad Prism 7 software. The scale bar is 500 μm. **D.** Quantification of alkaline phosphatase activity in mESC colonies using Image. The intensity of the red stain was measured using Image J (https://imagej.nih.gov) in 5 independent experiments. (For interpretation of the references to color in this figure legend, the reader is referred to the web version of this article.)

The morphology of mESC colonies reflects their self-renewal and pluripotency status. Dome-shaped colonies indicate undifferentiated and pluripotent cells, while flat and/or elongated colonies indicate loss of self-renewal and differentiation. Fig. 1A shows that mES cells exposed to BHPF at 0.5 ng/ml retain their dome-shaped morphology, while those exposed to higher concentrations from 1 ng/ml to 4 ng/ml exhibit altered morphology- flattened colonies, Fig. 1, C. In our experiments, media replenishment was performed every 2 days to maintain optimal culture conditions and nutrient availability for the mESCs. This step is critical as the media components get depleted, and the pH changes over time, which could affect the cells' physiology. Furthermore, it allows consistently delivering BHPF at the specified concentrations, ensuring that the observed effects are due to the compound and not influenced by media degradation or other time-dependent factors.

To further assess the impact of BHPF on the self-renewal capacity of mESC cells, we performed alkaline phosphatase staining, a marker of undifferentiated cells. Fig. 1C demonstrates that the alkaline phosphatase staining intensity decreases as the BHPF treatment concentration increases for 24 h. A quantitative analysis of the alkaline phosphatase stain reveals a significant reduction in the percentage of alkaline phosphatase-positive colonies, from 100 % in control to about 60 % in cultures exposed to 1 ng/ml to 4 ng/ml of BHPF.

**Fig. 2:**
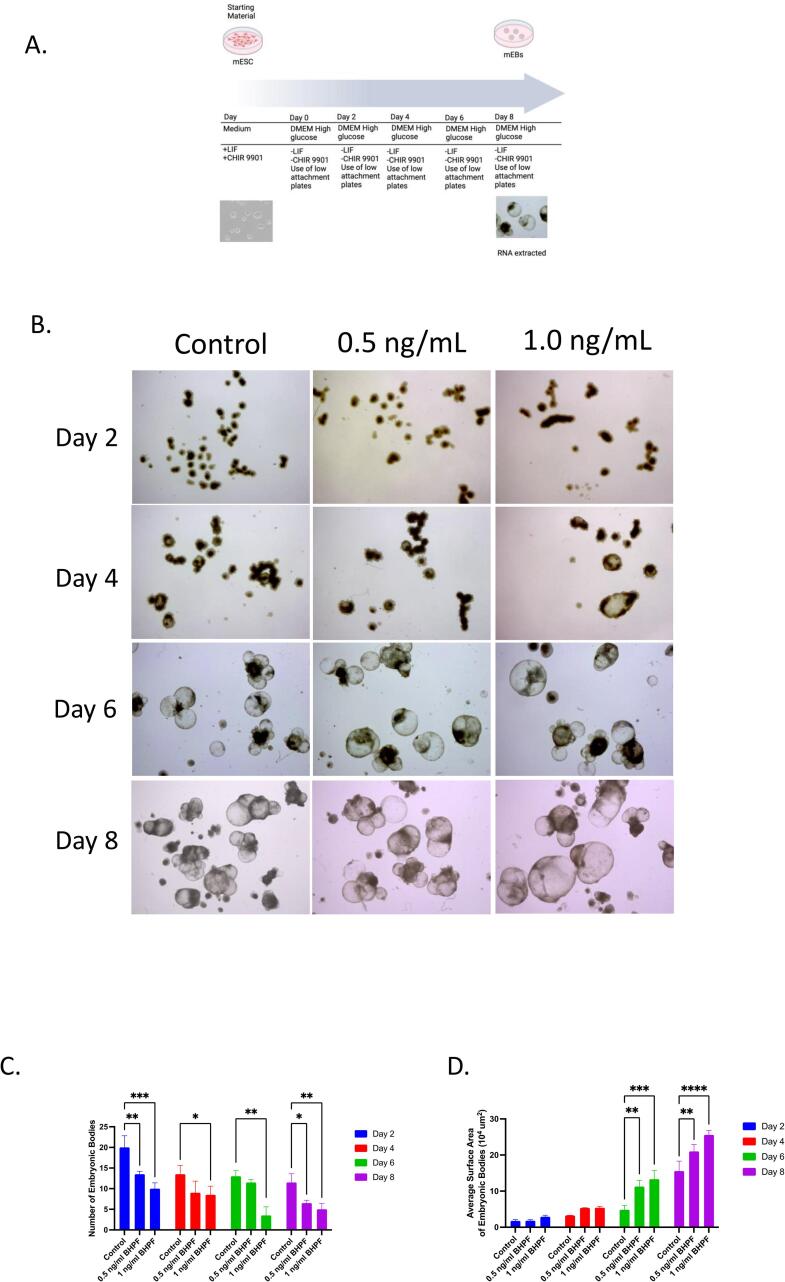
Mouse embryonic bodies followed for 8 days exposed to 0 ng/ml, 0.5 ng/ml, 1 ng/ml, 2 ng/ml, 3 ng/ml of BHPF for 24 h **A.** A graphical representation of the experimental methods adopted. **B.** morphology of embryoid bodies (EBs) from days 2, 4, 6, and 8. **B.** Bar graph of the total number of EBs for each day. **C.** A bar graph represents the total average area of the EBs for 8 days when exposed to BHPF. There were 3 independent experiments and 10 microscopical images were captured per independent experiment. The images were further analyzed with Image J software (https://imagej.nih.gov/). Data is shown as mean ± SEM. The micrometer bar represents 1 mm. *P <.05, **P ≤ 0.01, ***P ≤ 0.001 for the comparisons indicated.

To examine the effect of BHPF on the differentiation process of mESCs, we induced embryonic body (EB) formation by withdrawing leukemia inhibitory factor (LIF) from the culture medium. The control and BHPF-treated EBs were compared after culturing them without LIF for 8 days. We observed that on days 2, 4, 6, and 8, the number of EBs was reduced in the BHPF-exposed cultures compared to the control (Fig. 2B, C), indicating that BHPF might promote EB aggregation. Furthermore, we measured the size of the EBs and found that they were larger in the cultures treated with BHPF, especially on days 6 and 8, Fig. 2, D. These results suggest that BHPF affects the early stages of EB formation and alters their growth dynamics.

### 3.2 BHPF affects stress and toxicity pathway

Stress and Toxicity Pathway was utilized to investigate whether BHPF exposure triggers the expression of any of the 84 genes crucial for cellular adaptation to stress and toxic agents. Since different types of cells and tissues face various environmental challenges, such as oxidative damage, heat shock, osmotic imbalance, protein misfolding, and inflammation, a measure of these pathways would be beneficial. Our data reveals that when day 8, EBs were exposed to BHPF, only one gene, Il6, was differentially expressed, Fig. 4. Il6, a gene involved in the inflammatory response(Hirano, 2021), was approximately increased 3.8 times compared to the control.

**Fig. 3:**
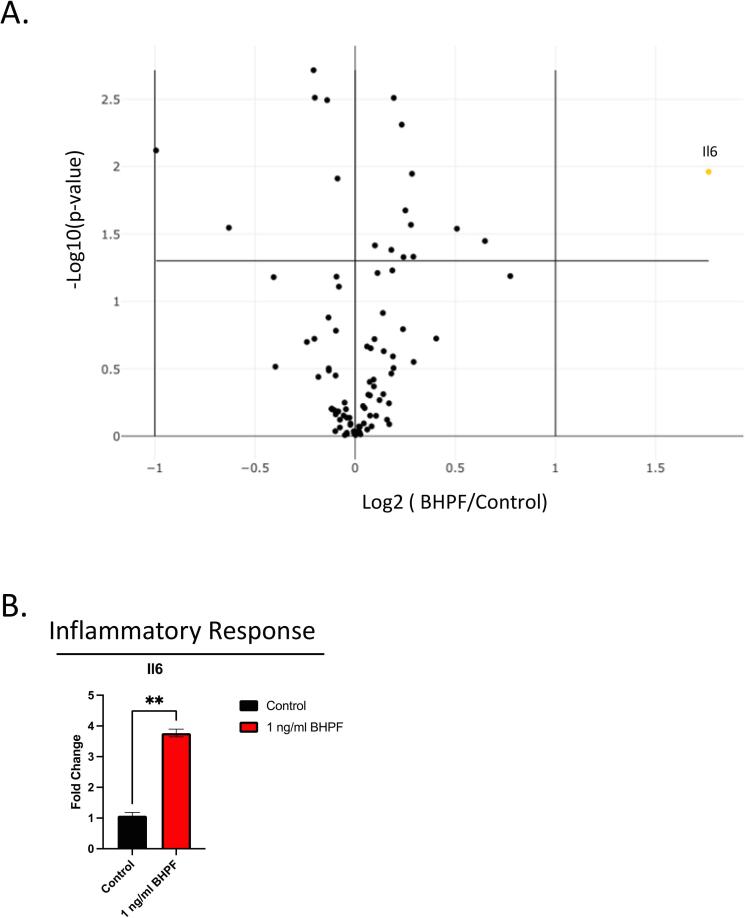
Differential expression of 84-genes related to stress and toxicity pathways in mouse day 8 mEBs exposed to 1 ng/ml of BHPF. **A.** A volcano plot displaying the log2-transformed fold change of each gene (y-axis) between BHPF-treated and control day 8 EBs versus the log2-transformed mean expression level (x-axis) of each gene across both groups. The horizontal black line indicates a 2-fold change cutoff. Genes with higher expression in BHPF-treated EBs are shown in yellow, while genes with lower expression are in blue. **B.** A bar plot shows the fold change of the only gene significantly differentially expressed, Il6, in the stress and toxicity pathways between BHPF-treated and control EBs. (For interpretation of the references to color in this figure legend, the reader is referred to the web version of this article.)

**Fig. 4:**
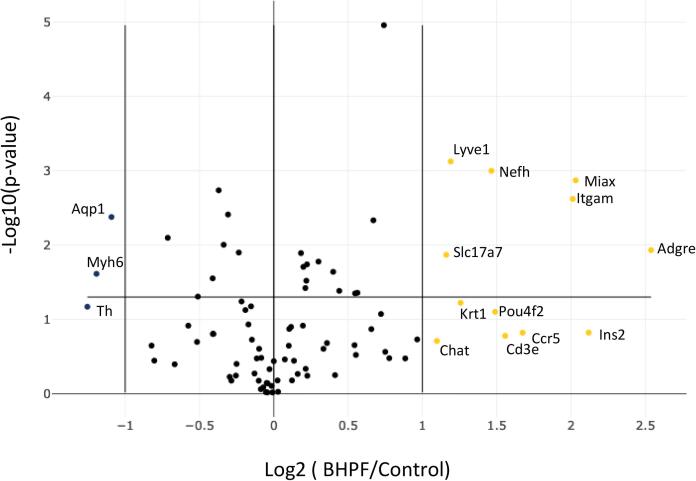
Expression profiles of 84-key genes associated with terminal differentiation in mouse day 8 mEBs exposed to 1 ng/ml of BHPF. A volcano plot showing the log2-transformed fold change (y-axis) of each gene between BHPF-exposed and control day 8 EBs versus the log2-transformed average expression (x-axis) of each gene in both groups. The horizontal black line marks a 2-fold change threshold. Genes with increased expression in BHPF-exposed EBs are indicated in yellow, while genes with decreased expression are in blue. (For interpretation of the references to color in this figure legend, the reader is referred to the web version of this article.)

### 3.3 BHPF affects terminal differentiation pathway

**Fig. 5:**
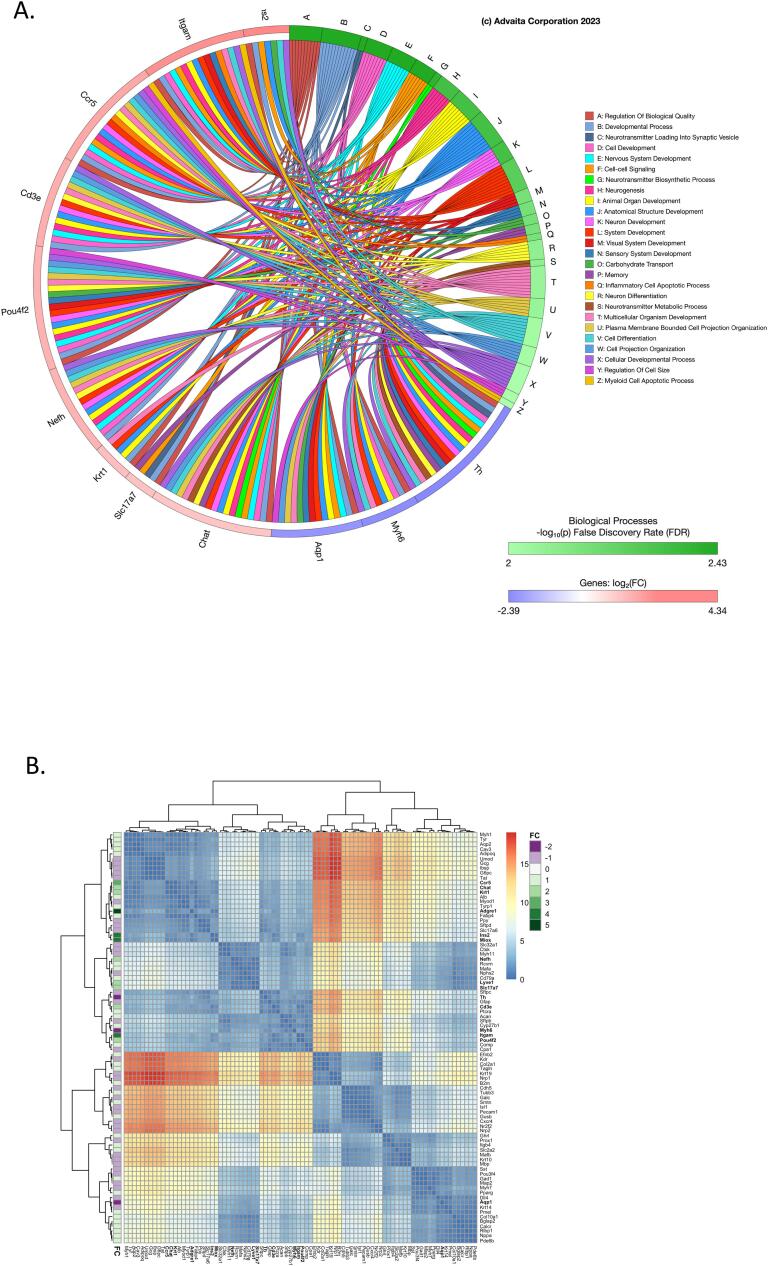
A. Chord diagram of the fold gene expression alterations from the terminal differentiation markers pathway and the top 20 associated Gene Ontology Biological Processes generated from the ipathway guide from Advaita Corporation. B. Heatmap of Euclidean distance between BHPF and control, showing clustering with complete linkage. The fold change values are shown in the sidebar, and the genes with absolute fold changes greater than 2 are in bold font.

Terminal differentiation pathway was utilized to measure the expression of 84 genes essential for recognizing distinct cell types. The generation of specific cell types from embryonic stem cell expression markers from 13 major organ/cellular types for cellular identification. Our data reveals that unlike in the other two pathways, BHPF increased the expression of 13 different genes and decreased the expression of 3 other genes, Fig. 4, Table 1. Our data suggest that BHPF at 1 ng/ml concentration in 8-day-old EBs affects endothelium-lymphatic endothelium subtype (Fig, 5, A), where Lyve1 gene is upregulated over two-fold; epithelium-keratinocyte epithelium subtype (Fig, 5, A), where Krt1 genes are also upregulated over 2-fold; pancreatic cells-beta cells subtype (Fig, 5, A), Ins2 gene is overexpressed over 4-fold; bone marrow - macrophages markers revealed that genes Adgre and Ccr5 are overexpressed approximately 6 and 3 times respectively. Bone marrow- -monocytes marker Itgam was also overexpressed more than 4-fold. Further analysis of the bone marrow early T-Cell development cell subtypes (Fig, 5, A) showed that Cd3e was overexpressed approximately 3 fold. In another tissue, the nervous system terminal differentiation markers were also altered. More specifically, mature neurons with the Nefh gene marker by approximately 3-fold, cholinergic neurons with the Chat gene marker by approximately 2-fold, dopaminergic neurons with Th marker were downregulated by more than 2-fold. Glutamatergic Slc17a7 marker were also upregulated by over 2-folds due to BHPF exposure when compared to control (Fig. 5, A). Another cell type, the retinal cells ganglion cell subtype (Fig. 5), was also upregulated where the Pou4F2 gene was increased in expression approximately 3-fold. Nephrons proximal tubule cells were also affected (Fig. 5, A), where the Aqp1 gene was approximately downregulated by 2-folds, and Miox, a marker for proximal tube cells, was upregulated by approximately 4 folds. Lastly, muscle cells-cardiomyocytes marker Myh6 was downregulated by over 2-folds when day 8 mEBs were exposed to BHPF for 1 ng/ml (Fig. 5, A). A heat map has been employed to delineate the spatial relationships among pairs of genes, predicated upon their Euclidean distances (Fig. 5, B). The color gradations within the heat map provide an intuitive illustration of these spatial affiliations. Specifically, a blue hue indicates that a given pair of genes are closely situated in Euclidean space, while a red hue denotes a more pronounced separation between them. The quantification of this separation is further expounded by the Average ΔCT values, which ascend to an approximate measure of 10. As a result, the maximum hypotenuse deduced from these values reaches up to 15, elucidating the magnitude of separation between gene pairs. Moreover, the graphical representation encompasses clustering along its peripheries, which demonstrates the similarity in behavioral patterns among the gene pairs in Euclidean space, upon juxtaposition against all other genes. Specifically, a pair of genes that are proximally clustered evince a congruent expression pattern across the entire gene ensemble. This clustering facet not only validates the spatial relationships depicted in the heat map but also enriches our understanding of the collective gene behavior within the scrutinized system, thereby providing a comprehensive insight into the multidimensional interactions that are at play.

**Table 1:** Significantly differentially expressed terminal differentiation markers (>2 fold).

Gene	Gene Name	Fold Regulation	Pathways
Ccr5	C-C chemokine receptor type 5	3.19	Bone Marrow Macrophages
Cd3e	CD3 antigen, epsilon polypeptide	2.94	Bone Marrow Early T-Cell Development
Chat	Choline acetyltransferase	2.14	Nervous System Cholinergic Neurons
Adgre1	Adhesion G protein-coupled receptor E1	5.81	Bone Marrow Macrophages
Ins2	Adhesion G protein-coupled receptor E1	4.34	Pancreatic Beta Cells
Itgam	Adhesion G protein-coupled receptor E1	4.03	Bone Marrow Monocytes
Krt1	Keratin 1	2.39	Epithelium Keratinocyte
Lyve1	Lymphatic vessel endothelial hyaluronan receptor 1	2.28	Endothelium Lymphatic
Miox	Myo-inositol oxygenase	4.09	Nephrons Proximal Tubule Cells
Nefh	Neurofilament, heavy polypeptide	2.76	Nervous System Mature Neurons
Pou4f2	Neurofilament, heavy polypeptide	2.81	Retinal Ganglion Cells
Slc17a7	Solute carrier family 17 (sodium-dependent inorganic phosphate cotransporter), member 7	2.24	Nervous System Glutamatergic Neurons
Aqp1	Solute carrier family 17 (sodium-dependent inorganic phosphate cotransporter), member 7	−2.13	Nephrons Proximal Tubule Cells
Myh6	Solute carrier family 17 (sodium-dependent inorganic phosphate cotransporter), member 7	−2.29	Muscle Cells Cardiomyocytes
Th	Solute carrier family 17 (sodium-dependent inorganic phosphate cotransporter), member 7	−2.39	Nervous System Dopaminergic Neurons

## 4 Discussion

In this study, we systematically examined BHPF's effects on mESCs and mEBs, focusing on morphology, stress pathways, and terminal differentiation. BHPF significantly altered mESC morphology and impacted 16 genes in terminal differentiation, consistent with its identified potential for developmental toxicity (Jin,Liu et al., 2022). This comprehensive analysis extends our understanding of BHPF's developmental toxicity and provides a foundation for future studies to elucidate the mechanisms and long-term effects of BHPF exposure.

We exposed mESCs to different concentrations of BHPF for 24 h and assessed their morphology, self-renewal capacity, and alkaline phosphatase activity. Our results show that BHPF affects the gross morphological features of mESCs and mEBs in a dose-dependent manner (Fig. 1, Fig. 2). BHPF exposure alters the colony morphology of mESCs from dome-shaped to flattened or elongated, indicating loss of pluripotency and differentiation (Fig. 1, A). BHPF also reduces the alkaline phosphatase activity of mESCs, a marker of undifferentiated cells (Fig. 2). Moreover, BHPF exposure decreases the number of mEBs formed from mESCs and increases their size, suggesting that BHPF promotes mEB aggregation.

PCR Array data indicated a 3.8-fold increase in Il6 expression in BHPF-exposed mEBs compared to controls (Fig. 3). This upregulation, in line with Il6′s known role as an inflammatory cytokine (Uciechowski and Dempke, 2020), suggests potential inflammation induction by BHPF. Examination of the terminal differentiation markers (Fig. 4) revealed that the expression of Lyve1, a marker associated with lymphatic endothelium development, was upregulated approximately 2-fold (Fig, 5A). Lyve1 encodes for lymphatic vessel endothelial hyaluronan receptor 1, a transmembrane protein involved in hyaluronan binding, lymphatic fluid homeostasis, and immune regulation (Banerji et al., 1999). Additionally, Lyve1 is expressed not only in lymphatic endothelial cells but also in certain tissue-resident macrophages (Jackson, 2003). Although the exact role of Lyve1 in development remains not fully understood, previous studies have indicated its involvement in processes such as angiogenesis (Karinen et al., 2022), lymphangiogenesis (Karinen et al., 2022), tissue remodeling (Karinen et al., 2022), and decidualization (Volchek et al., 2010). Notably, BHPF exposure has disrupted decidualization in both human and mouse models (Jin et al., 2022). Therefore, the upregulation of Lyve1 in response to BHPF exposure could affect lymphatic endothelial cells.

Another upregulated gene is Krt1, which encodes for keratin 1, a type II keratin protein that forms intermediate filaments with type I keratins in epithelial cells (Moll et al., 2008). Krt1 is a marker for the epithelium-keratinocyte epithelium subtype (Fig. 4, Fig. 5), where Krt1 gene expression was increased by more than two-fold in BHPF-treated mEBs versus the control. Krt1 is known to be involved in forming and maintaining the skin barrier by contributing to the structural integrity of keratinocytes and participating in the cornification process, indicating that BHPF may affect epithelial cell development (Coulombe and Omary, 2002). The Ins2 gene was overexpressed in pancreatic beta cells more than 4-fold (Fig. 5). Upregulation of the Ins2 gene may imply potential alterations in pancreatic beta cell function and insulin production (Melloul et al., 2002). Moreover, the bone marrow markers, Adgre and Ccr5, were upregulated (Fig. 4, Fig. 5), indicating a potential modulation of hematopoietic lineage, potentially impacting immunity (Geissmann et al., 2010).

Changes in the expression of nervous system terminal differentiation markers were observed, suggesting BHPF's influence on neuronal differentiation and maturation (Fig. 5, A). For instance, overexpression of Nefh and Chat could influence mature and cholinergic neurons, potentially affecting neuronal signaling. Contrastingly, it was observed that tyrosine hydroxylase Th gene expression was downregulated by approximately 2–fold Th gene expresses the rate-limting enzyme for the synthesis of catecholamine, which is responsible for the formation of L-DOPA (Molinoff and Axelrod, 1971). Catecholamines dopamine, epinephrine, and norepinephrine are crucial hormones and neurotransmitters in the central and peripheral nervous systems, originating from the catecholamine biosynthetic pathway(Daubner et al., 2011). The implications of this BHPF-induced Th downregulation may warrant further investigation to elucidate its impact on catecholamine biosynthesis and subsequent physiological processes.

Intriguingly, our data revealed an upregulation of the Pou4F2 gene, a known marker for retinal ganglion cells, suggesting potential implications for visual development (Badea and Nathans, 2004). This observation is consistent with recent findings in which eye malformation was reported in Chinese Medeka, a teleost fish, following exposure to BHPF at different concentrations for 105 days (Fan et al., 2023). The upregulation of Pou4F2 and the associated eye malformation highlight the need for further studies to explore the impact of BHPF on visual development. Altered expression of nephron markers Aqp1 and Miox was observed (Fig. 5), potentially implying renal impairment, a suggestion supported by earlier findings (Schnermann et al., 1998). In line with these findings, other mouse studies have documented liver injury following BHPF exposure (Yang et al., 2021). The implications of these observed changes in gene expression and their potential effects on renal and hepatic function warrant further exploration.

Finally, we observed a downregulation of the myh6 gene, a cardiac-specific marker encoding alpha-myosin heavy chain (α-MHC), a crucial component of the sarcomere in cardiac muscle cells, suggesting potential consequences for heart development and function (Ramírez and Padrón, 2004, Bersell et al., 2009). These findings align with previous research demonstrating downregulation of myh6 expression following BHPF exposure in zebrafish embryos (Mi et al., 2020). The observed changes in gene expression may indicate that BHPF exposure could disrupt normal cardiac development by altering the expression of genes central to cardiac morphogenesis and function.

Moreover, Gene Ontology (GO) terms serve as a critical framework for elucidating the roles of differentially expressed (DE) genes in various biological processes. Employing over-representation analysis and adjustments for multiple comparisons, we identified differentially expressed genes and mapped them to pertinent GO biological processes (Fig. 5A). These processes were then ranked and visualized using a chord diagram, which highlighted the top-scoring biological processes examined. The corroborative evidence from the ToxCast/Tox21 dataset strengthens our conclusions and lends external validity to our findings, where several common pathways with the ToxCast/Tox21 for BHPF (CASN 3236–71-3, DTXID5037731) dataset substantiate the role of BHPF in neurogenesis, neurotransmission, and neuron development (Table S1). These overlaps serve to validate our findings and extend the current understanding of BHPF's influence on neuroactivity. Uniquely, our study also explores pathways related to hippocampal neuron apoptotic processes, regulation of these processes, and neuromuscular junction development. These unique findings suggest a potential role for BHPF in developmental neurotoxicity. However, the emphasis on myeloid pathways sets our research apart from what is available in the ToxCast/Tox21 database. Upon assessing the effects of BHPF on embryonic bodies, our heatmap analysis revealed distinct spatial relationships among genes within the terminal differentiation pathway (Fig. 5B). The heatmap's blue hues encapsulate genes like Cd3e and Krt1, suggesting potential co-regulation or shared functionalities, possibly in T-cell development and keratinocyte differentiation upon BHPF exposure. The clustering, as revealed by dendrograms, accentuates gene sets that might collaboratively mediate the terminal differentiation events under BHPF influence. Their synchronized expression patterns postulate shared regulatory elements or pathways that BHPF predominantly impacts. The detailed heatmap analysis underscores specific genes and their potential roles in BHPF-mediated effects on embryonic body differentiation. These insights not only illuminate the molecular dynamics induced by BHPF but also lay the groundwork for deeper investigations into its cellular and molecular implications. Our investigation has opened up novel avenues for scholarly inquiry in the realm of myeloid cells. Therefore, future research could benefit from a targeted investigation into BHPF’s effects on myeloid cells, given its established role in neuroactivity and potential implications for immune response and tissue development.

Our observations indicate notable changes in gene expression, but these alterations are most significant in the context of embryonic body (EB) formation, a key focus of our study. While these findings enrich our understanding of the potential pathways affected by BHPF, it's crucial to approach these results with caution. Our study did not explore specific cell lineages, making these results indicative rather than conclusive. Nonetheless, the consistent results in EB formation provide valuable insights into the effects of BHPF at the cellular level.

Our study compares BHPF effects with those of other bisphenols like BPA, BPS, and BPAF, known endocrine disruptors affecting multiple biological systems (Vandenberg et al., 2007). BPS, another BPA substitute, also displays endocrine-disrupting potential, affecting reproductive development, metabolic processes, and brain development (Rochester and Bolden, 2015). Although less common, Bisphenol AF (BPAF) has been identified as potentially a stronger endocrine disruptor than BPA, primarily affecting reproductive health, thyroid function, and metabolic processes (Li et al., 2012). Bisphenols have unique effects on development, and these compounds' potential health risks are not yet fully comprehended. Ongoing investigation into their action mechanisms and long-term consequences is imperative to safeguard human health and development. These results highlight the broad range of developmental processes that may be influenced by BHPF exposure and emphasize the need for further investigation into the mechanisms and long-term consequences of BHPF toxicity. Our study provides valuable insights into the potential developmental toxicity of BHPF, indicating that its exposure leads affected key genes involved in neurogenesis, cell adhesion, signaling, inflammation, lymphatic endothelium development, epithelial differentiation, pancreatic beta cell function, hematopoiesis, and cardiac development. These results underscore the need to further explore the mechanisms and long-term effects of BHPF toxicity.

## 5 Conclusion

In summary, our study provides preliminary insights into the potential developmental toxicity of BHPF using mouse embryonic stem cells (mESCs) and mouse embryoid bodies (mEBs) as a model system. BHPF exposure led to observable alterations in mESCs and mEBs, suggesting changes in pluripotency and differentiation processes. Furthermore, BHPF exposure disrupted the expression of key genes involved in neurogenesis, cell adhesion, signal transduction, inflammation, lymphatic endothelial development, epithelial differentiation, pancreatic beta cell function, hematopoiesis, and cardiac development. However, it is important to note that our study focused on the early stages of cell differentiation, primarily assessing EB formation. It did not directly assess terminal differentiation of specific cell types. Therefore, while our findings indicate the potential effects of BHPF on developmental processes, further studies are needed to directly investigate the impact of BHPF on terminal differentiation and to comprehensively assess the developmental toxicity of BHPF.

## CRediT authorship contribution statement

**Aidan J. McLaughlin:** Formal analysis, Visualization. **Anthony I. Kaniski:** Formal analysis. **Darena I. Matti:** Formal analysis. **Nicodemus C. Monear:** Formal analysis. **Jessica L. Tischler:** Conceptualization. **Besa Xhabija:** Conceptualization, Methodology, Validation, Investigation, Funding acquisition, Formal analysis, Writing – original draft, Visualization, Supervision, Project administration, Resources.

## Declaration of Competing Interest

The authors declare that they have no known competing financial interests or personal relationships that could have appeared to influence the work reported in this paper.

## Data Availability

Data will be made available on request.
