# Supplementary material for: Fluorene-9-bisphenol affects the terminal differentiation of mouse embryonic bodies
Source: Curr Res Toxicol. 2023 Nov 2;5:100133. doi: 10.1016/j.crtox.2023.100133 (PMC10641737; doi:10.1016/j.crtox.2023.100133)
Supplement: Supplementary data 1 [file mmc1.docx]

Table S1. Common Biological processes between our data and the ToxCast/Tox21 dataset for BHPF (CASN 3236-71-3, DTXID5037731)

| ToxCast/Tox21 dataset for BHPF (CASN 3236-71-3, DTXID5037731) | | Biological Processes from our data with significant p-value |
| --- | --- | --- |
| Neuroactivity | neurotransmitter loading into synaptic vesicle, neurotransmitter biosynthetic process, neurogenesis, neuron development, neuron differentiation, neurotransmitter metabolic process, generation of neurons, neuron projection development, regulation of neurotransmitter levels, sequestering of neurotransmitter, positive regulation of hippocampal neuron apoptotic process, establishment of synaptic specificity at neuromuscular junction, neurofilament bundle assembly, aminergic neurotransmitter loading into synaptic vesicle, peripheral nervous system neuron axonogenesis, hippocampal neuron apoptotic process, regulation of hippocampal neuron apoptotic process, positive regulation of oxidative stress-induced neuron death, cell morphogenesis involved in neuron differentiation, neuron projection extension, neurofilament cytoskeleton organization, neuron projection morphogenesis, peripheral nervous system neuron differentiation, peripheral nervous system neuron development, regulation of neuron differentiation, neurotransmitter transport, neuron cellular homeostasis, neuromuscular synaptic transmission, regulation of oxidative stress-induced neuron death, neuron death in response to oxidative stress, neuron death, regulation of neurogenesis, neuromuscular junction development, neuron projection regeneration, regulation of neuron projection development, neuromuscular process controlling balance, positive regulation of neuron apoptotic process, negative regulation of neuron differentiation, positive regulation of neuron differentiation, positive regulation of neuron death, negative regulation of neuron projection development, negative regulation of neurogenesis, neuromuscular process, neuron projection guidance, regulation of neuron apoptotic process, positive regulation of neurogenesis, neuron apoptotic process, regulation of neuron death | |
| DNA Binding | Positive regulation of transcription regulatory region DNA binding, Regulation of transcription regulatory region DNA binding, Positive regulation of DNA binding, Regulation of DNA binding | |
| Cell Adhesion Molecules | Cell adhesion | |
| Cytokine | Positive regulation of cytokine production, Positive regulation of cytokine-mediated signaling pathway, Cellular response to cytokine stimulus, Response to cytokine | |
| Kinase | Positive regulation of phosphatidylinositol 3-kinase activity, Regulation of phosphatidylinositol 3-kinase activity, Positive regulation of lipid kinase activity, Transmembrane receptor protein tyrosine kinase signaling pathway | |
| Growth Factor Receptor | Growth | |
